# Development of a Diagnostic Prediction Model for Post‐Stroke Cognitive Impairment in Acute Large Vessel Occlusion Stroke Using Multimodal MRI and PET/CT: A Study Protocol

**DOI:** 10.1002/brb3.70613

**Published:** 2025-06-10

**Authors:** Junhao Li, Yuding Luo, Pingchuan Liu, Jiali Zhang, Chuanxi Duan, Hai Xiong, Maoxia Li, Binyang Zhang, Lu Li, Lulu Gong, Yupeng Niu, Bo Zheng, Jian Wang

**Affiliations:** ^1^ Department of Neurology The Affiliated Hospital, Southwest Medical University Luzhou China; ^2^ Department of Neurology Ya'an People's Hospital Ya'an China; ^3^ North Sichuan Medical College Nanchong China; ^4^ Department of Radiology Ya'an People's Hospital Ya'an China; ^5^ Sichuan Agricultural University Ya'an China

**Keywords:** post‐stroke cognitive impairment, magnetic resonance imaging, positron‐emission tomography, machine learning, endovascular treatment

## Abstract

**Objective:**

Stroke is a leading cause of morbidity and disability worldwide. Post‐stroke cognitive impairment (PSCI) significantly affects long‐term prognosis in acute anterior circulation large‐vessel occlusion stroke (LVO‐AIS). This study aims to develop a PSCI prediction model integrating multimodal imaging, demographic, and clinical data collected during hospitalization.

**Methods and Design:**

This single‐center, prospective cohort study will enroll 379 anterior circulation LVO‐AIS patients undergoing emergency endovascular treatment (EVT) within 24 h of symptom onset. Participants will be categorized into PSCI and non‐PSCI groups and followed up at 90 and 180 days post‐procedure. Primary outcomes include Montreal Cognitive Assessment scores at 3 and 6 months, with the modified Rankin Scale as a secondary outcome. Baseline imaging data will be processed using 3D Slicer for MRI and PET/CT standardization, registration, and feature extraction. Machine learning models will be developed using these imaging features combined with demographic and clinical data and evaluated via metrics such as the area under the receiver operating characteristic curve, precision, and recall. Analyses will be conducted in a blinded manner.

**Conclusion:**

This study will develop a PSCI prediction model based on multimodal imaging and clinical data in EVT‐treated LVO‐AIS patients, providing a tool for early diagnosis and personalized interventions. While limited to a single‐center, future multicenter validation is necessary to establish its generalizability and clinical utility.

## Graphical abstract

This study develops a machine learning‐based model to predict post‐stroke cognitive impairment (PSCI) in patients with anterior circulation large‐vessel occlusion who received endovascular treatment (EVT) within 24 h, using multimodal MRI and PET/CT imaging combined with clinical data for early risk stratification.

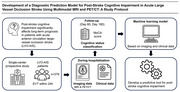

## 1 Introduction

Acute ischemic stroke (AIS) remains a leading cause of high morbidity and mortality worldwide, posing a significant threat to global health. Among AIS cases, large vessel occlusion (LVO) accounts for approximately 30% and is associated with markedly poorer clinical outcomes compared to non‐LVO cases (Martin et al. 2024; Lakomkin et al. 2018). A nationwide prospective real‐world registry study revealed that 77.9% of AIS‐LVO patients undergoing endovascular treatment (EVT) presented with anterior circulation occlusion (Jia et al. 2021). Encouragingly, the therapeutic window for EVT in patients with acute anterior circulation LVO has been extended to 24 h after symptom onset, significantly reducing disability and mortality rates compared to pharmacological therapy alone (Nguyen et al. 2024). However, despite achieving effective recanalization in 90% of EVT‐treated patients, only 50% attain favorable outcomes (Goyal et al. 2016). Challenges during the recovery phase, such as in‐stent restenosis, poor reperfusion caused by the no‐reflow phenomenon, and post‐stroke cognitive impairment (PSCI), continue to adversely impact long‐term prognosis (Zhang et al. 2024; Dowling et al. 2024; Luo et al. 2024). PSCI, characterized by cognitive decline occurring 3–6 months after stroke, affects at least one‐third of stroke survivors but often presents with subtle symptoms (Assayag et al. 2022). PSCI differs significantly from other post‐stroke complications, such as motor dysfunction, sensory impairment, and mood disorders, in both its clinical presentation and detection. Unlike motor and sensory deficits, which typically manifest acutely and are readily assessed by neurologists, PSCI often has an insidious onset and gradual progression, making early identification particularly challenging (El Husseini et al. 2023). Cognitive symptoms are frequently masked by physical disabilities or mistaken for normal aging processes, further complicating diagnosis (Huang et al. 2022). Moreover, most patients do not proactively report cognitive issues, and although tools, such as the Montreal Cognitive Assessment (MoCA) and MMSE, can assist in diagnosing PSCI, interventions based on their findings are often initiated too late to be clinically effective (Jacquin et al. 2014; Wang et al. 2021). For patients undergoing EVT, PSCI is more likely to have a substantial impact on long‐term social participation than motor or sensory deficits (Nguyen et al. 2024; Dowling et al. 2024). These challenges underscore the urgent need for systematic and targeted cognitive screening, as well as the development of more sensitive diagnostic tools. Thus, the accurate identification of high‐risk stroke survivors for PSCI is crucial for improving outcomes.

CT is advantageous for its speed and ease of operation. However, compared to CT, MRI offers superior tissue resolution and higher sensitivity to ischemic lesions. MRI enables earlier and more accurate identification of the ischemic penumbra and infarct core, providing comprehensive information, particularly in cases where CT fails to reveal significant abnormalities (Von Kummer and Dzialowski 2007). PET/CT, through the study of brain function, metabolic activity, and molecular biological processes, offers a unique imaging approach. By utilizing radiotracers such as ^18^F‐FDG, PET/CT can predict the risk of progression from normal cognition to dementia, highlighting its value in assessing neurodegenerative and ischemic conditions (Kato et al. 2016). However, previous studies have demonstrated significant limitations associated with using a single imaging modality for PSCI prediction. Although structural MRI can reveal morphological changes such as brain atrophy and white matter lesions, newly identified ischemic lesions (as detected by MRI) have not reliably predicted the development of cognitive impairment, suggesting that the sensitivity and specificity of structural imaging alone are limited (Molad et al. 2019; Biesbroek et al. 2022). In contrast, PET/CT can detect early metabolic dysfunction, but its spatial resolution is lower and its anatomical localization capability is limited (Aiello et al. 2019). Therefore, integrating the high‐resolution anatomical information provided by MRI with the metabolic functional insights from PET/CT can complement the limitations of each modality, thereby enhancing the accuracy of early PSCI prediction and improving risk stratification.

Machine learning (ML), an emerging tool in medical predictive modeling, applies computer science and statistics to support decision‐making and advance precision medicine. In PSCI research, ML efficiently handles complex high‐dimensional data such as clinical variables, biomarkers, and radiomic features by extracting key features, reducing redundancy, lowering dimensionality, and preventing overfitting. It also integrates multimodal data to enhance predictive accuracy (Li et al. 2023; Shurrab et al. 2024). Li et al. (2023), through a systematic review and meta‐analysis, demonstrated that ML methods can integrate multiple variables—including age, years of education, infarct volume, lesion location, and vascular risk factors—with greater predictive accuracy than traditional linear models, achieving a *c*‐index of 0.82. This finding highlights the potential of ML techniques to synthesize multidimensional clinical indicators and enhance the accuracy of cognitive risk prediction.

In this study protocol, we described the development of a PSCI prediction model for patients with acute anterior circulation LVO stroke who undergo EVT within 24 h of admission based on MRI and PET/CT imaging data, combined with demographic and clinical information.

## 2 Participants and Methods

### 2.1 Study Design

**FIGURE 1:**
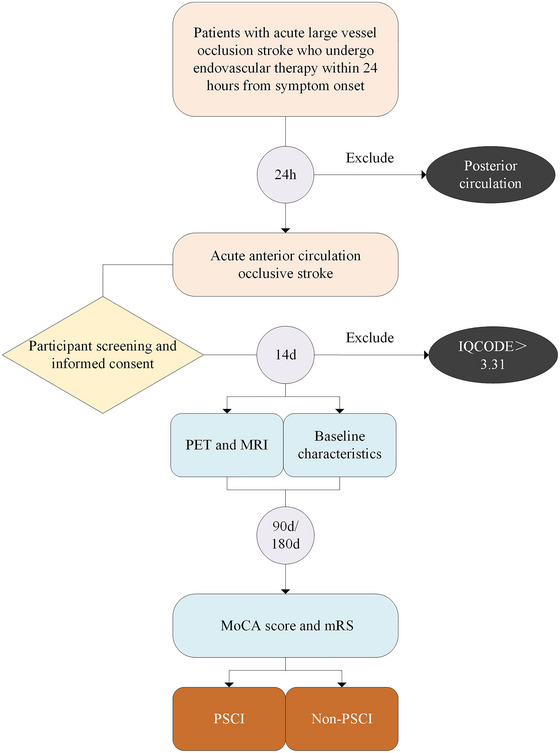
Sample selection and grouping. IQCODE, informant questionnaire on cognitive decline in the elderly; MoCA, Montreal Cognitive Assessment; mRS, modified Rankin Scale; PSCI, post‐stroke cognitive impairment.

This study is a single‐center, prospective, endpoint‐blinded clinical cohort study. PSCI is designated as the primary outcome, with patients who achieved vascular recanalization after emergency EVT within 24 h of symptom onset being stratified into PSCI and non‐PSCI groups. The follow‐up period is standardized to 180 ± 7 days. During hospitalization, data on cerebral glucose metabolism and MRI lesion distribution will be collected, alongside demographic and clinical information extracted from the case report system. Multivariate analyses will be conducted to investigate the associations between various indicators and the occurrence of PSCI in LVO‐AIS patients. Furthermore, this study aims to develop a PSCI prediction model by integrating radiomics with ML, evaluating its clinical applicability and potential utility. A detailed study workflow is illustrated in Figure 1.

This study was approved by the Ethics Committee of Ya'an People's Hospital (Approval No. 2023031) and registered with the Chinese Clinical Trial Registry (Registration No. ChiCTR2400093035, link: (https://www.chictr.org.cn/showproj.html?proj=223940). The study adheres to the principles of the Declaration of Helsinki.

### 2.2 Inclusion and Exclusion Criteria

Participants were recruited from the stroke unit of the Neurology Department at Ya'an People's Hospital and included patients with acute anterior circulation large‐vessel occlusion who underwent EVT within 24 h of symptom onset. Inclusion criteria were as follows: (1) Age >18 years; (2) symptom onset within 24 h, confirmed by CTA as acute anterior circulation LVO involving the intracranial internal carotid artery, middle cerebral artery (M1/M2), or anterior cerebral artery (A1/A2); (3) successful recanalization after emergency EVT (modified thrombolysis in cerebral infarction [mTICI] grade 2b‐3); (Nguyen et al. 2024) (4) with the ability to undergo the MoCA; and (5) provision of written informed consent by the patient or their family, with the capacity to complete all required examinations and follow‐ups. Exclusion criteria included (1) pre‐existing cognitive impairment prior to stroke; (2) admission NIHSS score >25 (scale range: 0–42, higher scores indicate more severe symptoms) or mRS score >2 (scale range: 0–6, higher scores indicate greater functional dependency) (Nguyen et al. 2024); (3) NIHSS item 1a >1 (scale range: 0–3, higher scores indicate greater impairment of consciousness); (4) history of AIS or transient ischemic attack (TIA); (5) presence of intracranial aneurysms or arteriovenous malformations; (6) implanted cardiac pacemakers or other non‐removable metallic devices; (7) diagnosis of diabetes mellitus; (8) claustrophobia, inability to remain still during imaging, or other conditions preventing successful completion of examinations; or (9) pregnant or breastfeeding women.

### 2.3 Data Collection

Baseline data for patients were collected from the case report system and included the following: age, sex, years of education, presence of atrial fibrillation, blood pressure, smoking and alcohol consumption status, and routinely available biochemical indicators such as blood glucose, triglycerides, low‐density lipoprotein, homocysteine, uric acid, and creatinine (Lee et al. 2023).

For cognitive assessment, the 16‐item version of the informant questionnaire on cognitive decline in the elderly (IQCODE) was used to evaluate patients’ pre‐stroke cognitive status through informant interviews. Each item is rated from 1 (much improved) to 5 (much worse), and the average score across all items is calculated, with higher scores indicating worse cognitive function. An IQCODE score of ≥3.31 was defined as indicative of pre‐stroke cognitive impairment. PSCI was assessed by a professionally trained neurologist at 90 ± 7 and 180 ± 7 days post‐discharge using the MoCA. The MoCA evaluates seven cognitive domains, with a maximum score of 30 points; lower scores indicate poorer cognitive function. The assessment takes approximately 15–20 min to complete. PSCI was defined as a MoCA score <22, with an additional point added for patients with less than 12 years of education (Wei et al. 2023). The Chinese versions of the IQCODE and MoCA scales used in this study have been validated in prior research for their reliability and validity (Dong et al. 2021; Li et al. 2012).

MRI imaging was performed using a Discovery MR750 scanner (GE Healthcare) with an eight‐channel head coil. The following sequences were acquired for all participants: T1‐weight: TR = 2528.3 ms, TI = 796 ms, TE = 24 ms, matrix = 288 × 224, FOV = 240 mm × 192 mm, thickness = 5 mm, number of layers = 20 layers; T2‐weight: TR = 4905 ms, TE = 111 ms, matrix = 416 × 208, FOV = 240 mm × 240 mm, thickness = 5 mm, number of layers = 20 layers; T2‐FLAIR: TR = 8000 ms, TI = 2350 ms, TE = 120 ms, matrix = 320 × 192, FOV = 230 mm × 230 mm, thickness = 5 mm, number of layers = 20 layers; susceptibility weighted imaging (SWI): TR = minimum, TE = 45.0 ms, flip angle = 15°, matrix = 384 × 320, FOV = 240 mm × 216 mm, layer thickness = 2 mm, number of layers = 40; diffusion weighted imaging (DWI): *b* value = 1000, TR = 4880 ms, TE = minimum, matrix = 130 × 160, FOV = 240 mm × 240 mm, layer thickness = 5 mm, number of layers = 20; diffusion tensor imaging (DTI): number of diffusion directions = 30, TR = 8000 ms, TE = minimum, matrix = 128 × 130, FOV = 240 mm × 240 mm, layer thickness = 5 mm, number of layers = 29. To ensure the quality and reproducibility of imaging data, multiple standardization and artifact control measures were implemented during MRI acquisition and preprocessing. During scanning, foam cushions were used to stabilize the participant's head and minimize motion artifacts. Following acquisition, all MRI sequences were first registered to the T1‐weighted images and subsequently standardized to the MNI152 template space using affine and nonlinear transformations. For DWI and DTI data, additional motion correction procedures were applied. Image quality control was performed through a combination of manual visual inspection and automated assessment tools such as MRIQC. Datasets exhibiting significant artifacts, such as ghosting or susceptibility distortions, were excluded. These measures were intended to reduce data variability and enhance the stability and reproducibility of radiomic feature extraction (Provins et al. 2023).

For PET/CT imaging, ^18^F‐FDG was produced using a Sumitomo medical cyclotron with a radiochemical purity >95%. Elevated blood glucose can reduce ^18^F‐FDG uptake in the brain, leading to underestimated glucose metabolism and reduced accuracy of quantitative image analysis (Viglianti et al. 2017). Therefore, patients fasted for more than 6 h prior to the scan and were instructed to rest quietly in a dimly lit environment after intravenous administration of ^18^F‐FDG. Brain PET/CT scans were performed 45 min post‐injection using a Siemens Biograph mCT scanner (64‐slice CT). The procedure included an initial brain CT scan for attenuation correction of PET/CT data, followed by a 10‐min 3D brain emission scan. PET/CT data were reconstructed using the ordered‐subset expectation maximization (OSEM) algorithm to generate cross‐sectional, coronal, and sagittal images of the brain.

### 2.4 Statistical Analysis

For sample size estimation, the formula was based on Riley et al. (2020) guidelines for binary outcome prediction models. The calculation utilized the “pmsampsize” package in R. The *c*‐statistic was set at 0.82 (*c*‐statistic = 0.82) based on Chander et al.’s study (Chander et al. 2017), which reported an AUC of 0.82 for similar prediction models. The prevalence of PSCI was set at 44% (prevalence = 0.44), according to the findings of Lo et al. (2019). The number of candidate predictors was set to 15 (parameters = 15), and the shrinkage factor was set to 0.8 (shrinkage = 0.8). The final calculated sample size was 379 participants, with 265 (70%) allocated to the training set and 114 (30%) to the validation set.

Statistical analyses were conducted using IBM SPSS Statistics 27.0, whereas data visualization was performed with GraphPad Prism 10. Continuous variables following a normal distribution were expressed as mean ± standard deviation (*X̅* ± SD), whereas non‐normally distributed variables were presented as median and interquartile range [M (IQR)]. Comparisons of continuous variables between groups were performed using the independent samples *t*‐test or the Wilcoxon rank‐sum test, as appropriate. Categorical variables were expressed as percentages, and comparisons between groups were conducted using the *χ* ^2^ test or Fisher's exact test.

The Benjamini–Hochberg method was used to control the false discovery rate (FDR). Bonferroni correction was applied as a supplementary validation for existing comparison results. Differences in AUC between groups were assessed for statistical significance using the DeLong test. To further elucidate patterns in feature distribution, stratified analysis and feature heatmaps were used to visually present the distribution differences of key features between the PSCI and non‐PSCI groups. All statistical tests were two‐sided, with the significance level set at *α* = 0.05.

To minimize the potential impact of confounding factors such as age, education level, and stroke severity on outcomes, specific control strategies were implemented in both traditional statistical analyses and ML modeling. In traditional statistical analysis, multivariable logistic regression models were constructed with these variables included as covariates. During ML model development, demographic and clinical variables were systematically incorporated into the feature set to adjust for their potential influence on predictive performance (Lee et al. 2023). These measures were designed to reduce confounding bias and enhance the robustness and generalizability of the study findings.

### 2.5 Machine Learning

#### 2.5.1 Image Processing and Feature Extraction

Both MRI and PET/CT imaging data were acquired within 14 days of hospitalization. The imaging data were preprocessed using 3D Slicer software, including standardization, registration, and normalization. MRI images were registered to the T1‐weighted sequence as the reference, standardized to the MNI152 template space, and resampled to a voxel size of 3 mm^3^. Regions of interest (ROIs) were delineated using 3D Slicer modules, with interpretations performed by two senior radiologists. To enhance segmentation consistency and accuracy, a deep learning‐based U‐Net model was employed for ROI segmentation. ROIs included gray matter, white matter, ventricles, and lesion areas. Radiomics features were extracted using the Radiomics plugin, encompassing shape, texture, and intensity distribution features. The features were exported in a standardized CSV format for subsequent analysis. Feature selection was performed through univariate analysis to identify important variables, followed by dimensionality reduction using principal component analysis (PCA) and uniform manifold approximation and projection (UMAP) to minimize redundant features while retaining critical information (Wang et al. 2022; Lopes et al. 2021). PCA effectively captures global linear variance and enhances feature interpretability, whereas UMAP excels at preserving complex local nonlinear structures, facilitating better identification of sample heterogeneity. Compared to t‐SNE, UMAP offers superior performance in revealing deep clustering patterns in two‐dimensional spaces. By employing both dimensionality reduction techniques, the model balances global interpretability with the preservation of local structural information, allowing for a complementary and synergistic analysis of the data (Yang et al. 2021; Lazli et al. 2022).

#### 2.5.2 Model Construction

**FIGURE 2:**
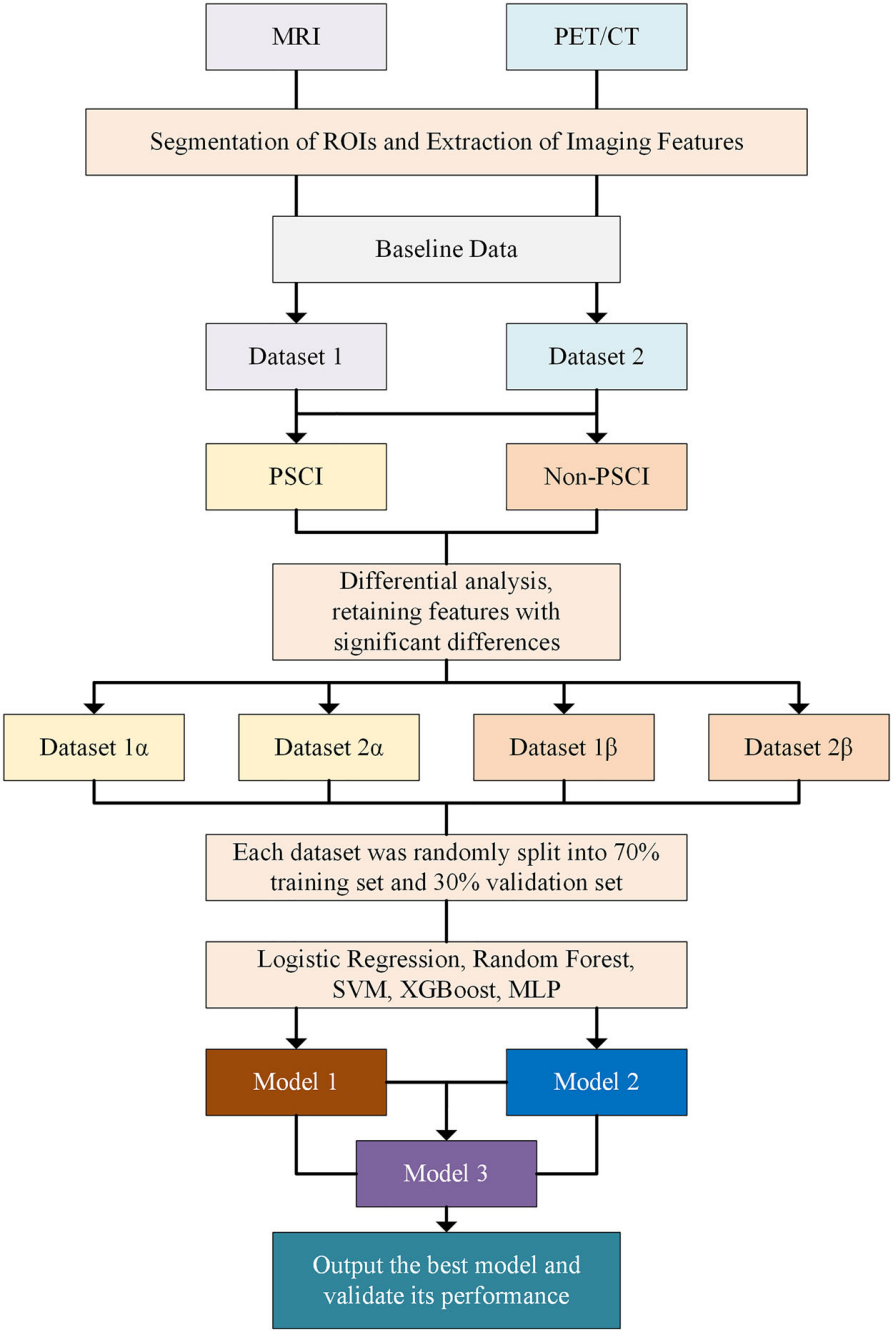
Predictive model construction. MLP, multi‐layer perceptron; PSCI, post‐stroke cognitive impairment; ROI, region of interest; SVM, support vector machine; XGBoost, extreme gradient boosting.

The model was built using R software version 4.3.3. The ML models were categorized into three groups: the MRI feature (Model 1), the PET/CT feature (Model 2), and the combined MRI and PET/CT feature (Model 3). Models 1 and 2 were developed using baseline data and implemented through logistic regression, random forest, support vector machine (SVM), extreme gradient boosting (XGBoost), and multi‐layer perceptron (MLP). Model 3 was constructed by stacking the outputs of Models 1 and 2. Model training employed fivefold cross‐validation to ensure robustness, with performance evaluated on the validation set using metrics, including the AUC–ROC curve, precision, recall, F1 score, 95% confidence interval, and Brier score. Shapley additive explanations (SHAP) was used to analyze the contribution of each feature to the prediction results, and confusion matrices and calibration curves were generated to assess classification performance and probability calibration. Detailed steps for model development are illustrated in Figure 2 (Lee et al. 2023; Lopes et al. 2021; Lee et al. 2022).

## 3 Discussion

Several recent advances have been made in the field of stroke research. A multicenter study from China highlighted that prehospital delays among stroke patients remain a significant issue, reporting a median onset‐to‐admission time of approximately 15 h for patients with AIS, with only about one‐quarter of patients arriving at the hospital within 3 h of symptom onset; this issue was further exacerbated during the COVID‐19 pandemic (Liao et al. 2024). At the molecular level, the PI3K/AKT signaling pathway has been identified as a key mediator of ischemic injury and represents a promising target for the development of neuroprotective therapies (Liu et al. 2025). In terms of therapeutic strategies, novel interventions are actively being explored. For example, simvastatin has been shown to reduce brain edema and neuronal apoptosis following experimental intracerebral hemorrhage by activating the VEGF‐C/VEGFR3/PI3K‐Akt signaling pathway and enhancing the glymphatic clearance system, thereby improving neurological outcomes (Liao et al. 2024). Additionally, the recent CARICH randomized controlled trial found that in the treatment of intracerebral hemorrhage, hematoma evacuation alone resulted in better functional outcomes—characterized by lower rates of severe disability and mortality—compared to combined hematoma evacuation and decompressive craniectomy (Zhang et al. 2024). These findings underscore the multifaceted efforts currently underway to improve stroke care, ranging from reducing prehospital delays to advancing molecular‐level neuroprotective strategies and optimizing acute‐phase interventions.

Various neuroprotective strategies have been explored in intervention studies targeting PSCI. DL‐3‐*n*‐butylphthalide (NBP), a neuroprotective agent, has been shown to significantly improve cognitive outcomes in ischemic stroke models by reducing infarct volume and alleviating PSCI through anti‐inflammatory and antioxidant pathways (Zhang et al. 2023). Natural compounds such as mangiferin have also demonstrated efficacy in mitigating post‐stroke cognitive deficits; in cerebral ischemia‐reperfusion models, mangiferin reduced neurological deficit scores, decreased infarct volume, ameliorated anxiety‐ and depression‐like behaviors, and improved cognitive function, partially by correcting dysregulated lipid metabolism (Zhang et al. 2024). In addition to pharmacological approaches, vascular factors contributing to PSCI have attracted increasing attention. Hemodynamic impairment caused by intracranial atherosclerosis can lead to chronic cerebral hypoperfusion and subtle ischemic injury, which are strongly associated with a higher incidence of cognitive decline among stroke survivors. Notably, even with aggressive interventions such as intracranial stenting aimed at restoring cerebral perfusion, clinical trials have failed to demonstrate significant cognitive improvement, highlighting the complexity of vascular cognitive impairment and the need for innovative strategies targeting cerebral hypoperfusion (Chen et al. 2024; Wang et al. 2019).

EVT, as one of the primary treatment options, has been proven beneficial for neurological recovery in stroke patients (Nguyen et al. 2024). Compared to pharmacological treatment alone, EVT offers superior outcomes but comes with higher treatment costs. This has heightened expectations for favorable prognoses among patients, their families, and clinicians. Consequently, we designed this study to explore PSCI prediction models in this specific patient population through a single‐center investigation.

This study aims to collect cognitive and functional outcomes in patients with anterior circulation large‐vessel occlusion treated with EVT. Cognitive status is assessed using MoCA scores at 3 and 6 months post‐stroke as the primary outcome measure, whereas functional prognosis is evaluated using the mRS as the secondary outcome measure. Anterior circulation occlusion constitutes a significant proportion of AIS‐LVO cases. A retrospective study indicated that approximately 36% of patients with anterior circulation occlusion develop PSCI, which is associated with functional outcomes (De Rubeis et al. 2023). By prospectively collecting follow‐up data, this study enables preliminary statistical analysis to identify factors associated with PSCI in patients treated with EVT.

Our study represents a novel approach by prospectively collecting multimodal MRI and PET/CT imaging data from patients with anterior circulation occlusion treated with EVT. Using specialized image processing software, this design uniquely integrates structural MRI data reflecting lesion‐related structures with PET/CT data capturing cerebral metabolism. Combined with baseline patient data, advanced ML techniques are applied to analyze the extensive dataset and construct a PSCI prediction model tailored to the characteristics of the study population. Given that hyperglycemia can impair FDG uptake in brain tissue and interfere with the semiquantitative analysis of PET imaging (Viglianti et al. 2017), patients with diabetes were excluded from this study to ensure the accuracy of imaging data. Although diabetes is an important risk factor for PSCI (Filler et al. 2024), future research could consider including patients with well‐controlled blood glucose levels to further validate the findings. For MRI sequences, SWI was used to identify cerebral microbleeds, T2‐weighted, T2‐FLAIR, and DWI sequences were used to detect ischemic lesions, and DTI data, along with tractography, was applied to analyze brain networks (Mijajlović et al. 2017). Notably, microbleeds, which affect cerebral autoregulation, have been implicated in the pathogenesis of PSCI (Chi et al. 2020). Although CT is more cost‐effective and widely available in lower tier hospitals compared to MRI, prior studies have primarily focused on PSCI prediction models based on CT imaging data (Quinn et al. 2021). However, CT provides less clarity in visualizing infarct areas compared to MRI (Von Kummer and Dzialowski 2007). To ensure the innovation of the prediction model and the accuracy of lesion interpretation, this study employs multimodal MRI combined with PET/CT for feature extraction.

In accordance with guidance, a shrinkage factor of 0.8 was applied to reduce the risk of overfitting, corresponding to an acceptable level of 20% optimism in the estimated predictor effects (Riley et al. 2020). This threshold ensures at least 10 events per parameter, which supports model stability and generalizability. Although shrinkage alone may not entirely eliminate the possibility of overfitting, particularly in smaller samples, using a prespecified factor of 0.8 provides a conservative safeguard. On the basis of the anticipated model performance (*c*‐statistic = 0.82), outcome prevalence (44%), and number of candidate predictors (*n* = 15), the final sample size of 379 represents a reasonable balance between model complexity and control of overfitting. This strategy aligns with Riley et al. (2020) core recommendation that integrating shrinkage considerations into sample size planning helps reduce model optimism and improves external validity.

Although feature selection and dimensionality reduction were performed using standardized approaches, potential bias in feature selection cannot be completely eliminated, particularly given the high dimensionality of imaging data relative to sample size. Cross‐validation and the application of PCA and UMAP were employed to mitigate redundancy and improve model robustness. However, the risk of overfitting remains a concern, especially when using complex ML algorithms such as random forest, SVM, XGBoost, and MLP. To address this, fivefold cross‐validation was implemented during model development, and a shrinkage factor of 0.8 was incorporated into sample size calculation to control model optimism. Additionally, model calibration was assessed using calibration curves to evaluate probability estimates (Lee et al. 2023; Lopes et al. 2021; Lee et al. 2022).

In the current interdisciplinary environment, ML has been widely applied in medical research and clinical practice (Eshaghi et al. 2021; Shamir et al. 2015). PSCI, often overlooked despite its significant association with family and societal economic burdens, highlights the critical need for identifying at‐risk populations and implementing early interventions. Lee M et al. retrospectively collected data from AIS patients, including 1.5T and 3T MRI images, annotated lesion locations and extents, and assessed brain structures using the modified Fazekas score and Scheltens score. By integrating demographic and clinical data, they developed an ML‐based prediction model that demonstrated robust performance (AUC = 0.79) (Lee et al. 2023). In contrast, predictive models utilizing PET/CT have predominantly been applied in the context of Alzheimer's disease (AD) (Kaur et al. 2024). Recently, Lee R et al. developed a deep learning model using ^18^F‐FDG PET/CT data from AD patients. When transferred to a prospective AIS cohort, the model maintained strong predictive performance (AUC = 0.75), supporting the feasibility of ^18^F‐FDG PET/CT for PSCI prediction (Lee et al. 2022). Additionally, in a recent study, Godefroy et al. (2025) used amyloid PET/CT scans and identified a significant association between amyloid status and PSCI. These findings underscore the potential of advanced imaging modalities, including ^18^F‐FDG PET/CT and amyloid PET/CT, for developing accurate and clinically relevant PSCI prediction models.

Early models often relied on single‐modality data and traditional statistical methods, typically using only clinical variables or a single imaging technique, and primarily employed conventional approaches such as logistic regression or simple risk scores (Lee et al. 2023). For instance, prognostic scoring systems like the CHANGE and SIGNAL_2_ models, which were based on limited clinical and imaging features, demonstrated moderate predictive performance for PSCI, with AUC values ranging from approximately 0.74 to 0.83, highlighting the limitations associated with narrow feature dimensions (Lee et al. 2023). Our model, by contrast, leverages a multimodal dataset that integrates complementary imaging modalities (MRI and ^18^F‐FDG PET/CT) along with clinical variables, thereby offering a more comprehensive representation of each patient's post‐stroke brain status. Radiomic feature extraction combined with dimensionality reduction techniques (PCA and UMAP) was employed to capture subtle, high‐dimensional patterns that single‐modality approaches might overlook. Furthermore, we implemented advanced ML algorithms—including SVM, Random Forest, XGBoost, and MLP—as well as a stacked ensemble model, enabling the capture of complex, non‐linear interactions beyond the capabilities of traditional regression models. Notably, integrating diverse data types through ML has been shown to enhance predictive accuracy in identifying individuals at risk, consistent with the strong performance of our ensemble model (Shi et al. 2024). Importantly, this increase in model sophistication does not compromise interpretability; we incorporated SHAP‐based explainable AI techniques to evaluate feature importance, thereby allowing clinicians to understand which imaging or clinical factors most influence the model's predictions (Lee et al. 2023). Our proposed model ensures enhanced data richness, refined feature engineering, advanced modeling techniques, and robust interpretability, ultimately improving predictive accuracy and offering deeper insights into post‐stroke cognitive risk.

This study has certain limitations. External validation was not included, and the clinical feasibility of the developed prediction model will be assessed based on its initial performance. Future studies will involve larger prospective cohorts to further evaluate the clinical applicability of the model. As a single‐center study, the design is limited by geographical constraints. Selection bias may have been introduced, as patient demographic characteristics, clinical practice patterns, and institutional workflows may not fully represent the broader stroke population. Additionally, limitations in imaging equipment and technical parameters could further restrict the generalizability of the predictive model. These factors may contribute to model overfitting and reduce its external validity across diverse clinical settings. On the other hand, as a limitation of this single‐center study, no independent external dataset has yet been planned for validation. Therefore, the generalizability of the developed predictive model may be somewhat limited. Expanding to multicenter studies in the future could address issues of sample size and generalizability by incorporating independent external datasets from multiple centers to comprehensively validate and optimize model performance across broader clinical settings. Additionally, due to restrictions on tracer availability at our center, amyloid PET/CT scans were not included in this study. Incorporating amyloid imaging in future research could enhance the robustness and accuracy of the prediction model.

In clinical practice, effective integration of the PSCI prediction model developed in this study into routine workflows requires the selection of an appropriate risk threshold and corresponding management strategies. On the basis of epidemiological data, approximately 25%–50% of stroke survivors may experience cognitive impairment within several months after stroke (Chander et al. 2017). Therefore, this study proposes defining patients with a predicted probability of PSCI exceeding 30% as a high‐risk group. This threshold can be adjusted as needed to maintain moderate specificity while ensuring a screening sensitivity of 80%–90% (Salvadori et al. 2022). Given potential limitations in imaging resources in real‐world clinical settings, the MRI‐based model (Model 1) and the PET/CT‐based model (Model 2) were designed as alternative predictive solutions. Although these alternatives may slightly compromise predictive accuracy, they offer greater practical feasibility and facilitate broader adoption of the risk assessment tool across healthcare environments with varying resource availability. By leveraging accessible predictive indicators, the models enable risk stratification even in resource‐constrained contexts, thereby extending their applicability (Chen et al. 2023).

The optimal threshold will be appropriately adjusted during the course of the study based on the characteristics of the patient population and resource availability at our medical center, taking into account the local prevalence of PSCI as well as acceptable rates of false negatives and false positives. Moreover, the risk predictions generated by the model are intended to complement, rather than replace, clinical judgment: Even patients classified as low‐risk should undergo regular cognitive reassessment, as no predictive model is infallible. Before widespread implementation, prospective validation studies should be conducted, and healthcare professionals should receive appropriate training to ensure the tool is used accurately and responsibly across different clinical settings. By addressing these considerations, the predictive model could be effectively integrated into post‐stroke care pathways as a practical adjunctive tool to facilitate the early identification of high‐risk individuals and timely initiation of cognitive interventions.

## Author Contributions

**Junhao Li**: writing – original draft, methodology, data curation. **Yuding Luo**: methodology, writing – original draft, software. **Pingchuan Liu**: methodology, data curation, writing – review and editing. **Jiali Zhang**: writing – review and editing, supervision. **Chuanxi Duan**: writing – review and editing, methodology. **Hai Xiong**: methodology, writing – review and editing. **Maoxia Li**: investigation, writing – review and editing. **Binyang Zhang**: investigation, writing – review and editing. **Lu Li**: investigation, writing – review and editing. **Lulu Gong**: investigation, writing – review and editing. **Yupeng Niu**: methodology, software, writing – review and editing. **Bo Zheng**: conceptualization, supervision, writing – review and editing. **Jian Wang**: conceptualization, supervision, funding acquisition, project administration, resources, writing – review and editing.

## Conflicts of Interest

The authors declare no conflicts of interest.

## Peer Review

The peer review history for this article is available at https://publons.com/publon/10.1002/brb3.70613

## Data Availability

Research data are not shared.
